# Supplementary material for: Engineering the Steroid Hydroxylating System from Cochliobolus lunatus in Mycolicibacterium smegmatis
Source: Microorganisms. 2021 Jul 13;9(7):1499. doi: 10.3390/microorganisms9071499 (PMC8306143; doi:10.3390/microorganisms9071499)
Supplement: Supplementary file 1 [file microorganisms-09-01499-s001.zip › microorganisms-1284090-supplementary.pdf]

Figure S1

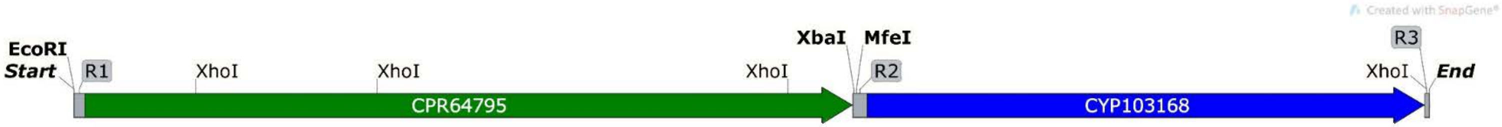

OPERON FAN
3692 pb

R1: ccggaattc**TGA**cc**TGA**gag**aaagggagTGATAA**
R2: **TGATAA**tctagacaattg**TGA**cc**TGA**gag**aaagggagTGATAA**
R3: **TAA**ctcgagcgg

Figure S2

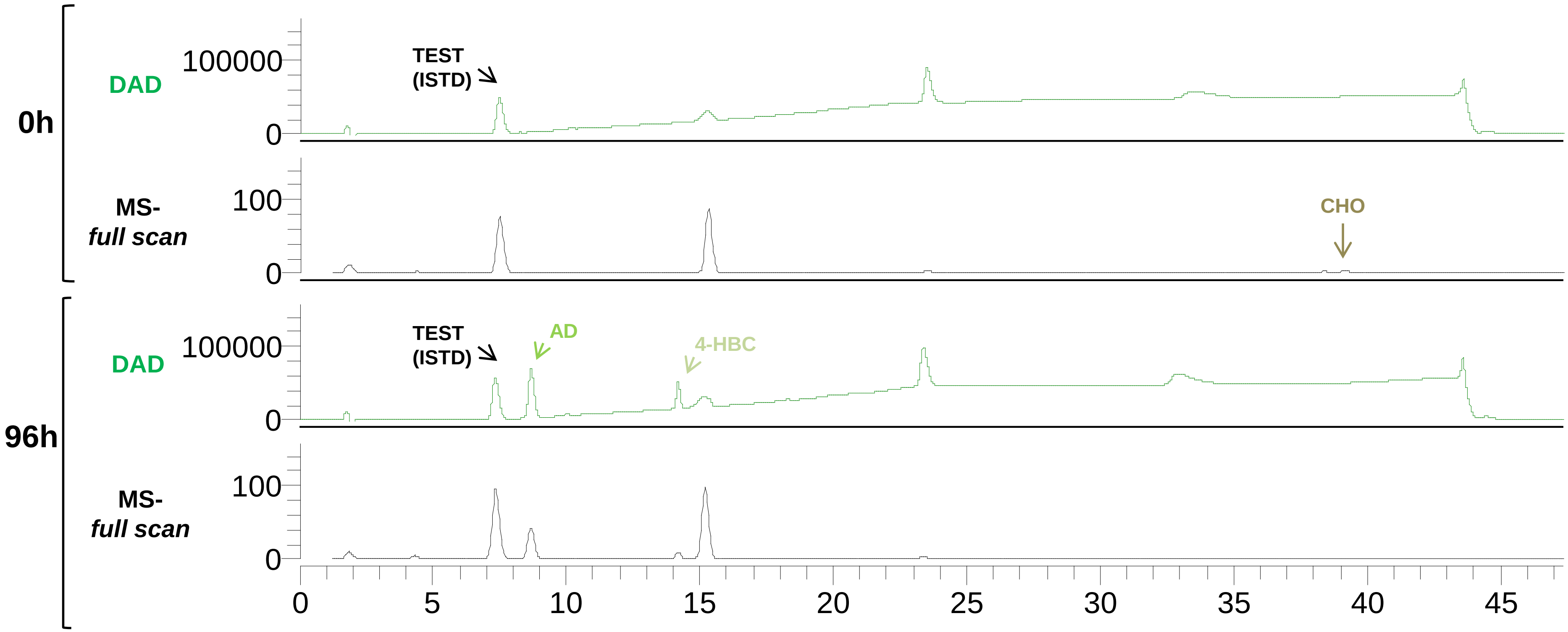

0h
DAD
100000
0
TEST
(ISTD)
MS-
full scan
100
0
CHO
96h
DAD
100000
0
TEST
(ISTD)
AD
4-HBC
MS-
full scan
100
0
0
5
10
15
20
25
30
35
40
45

Figure S3

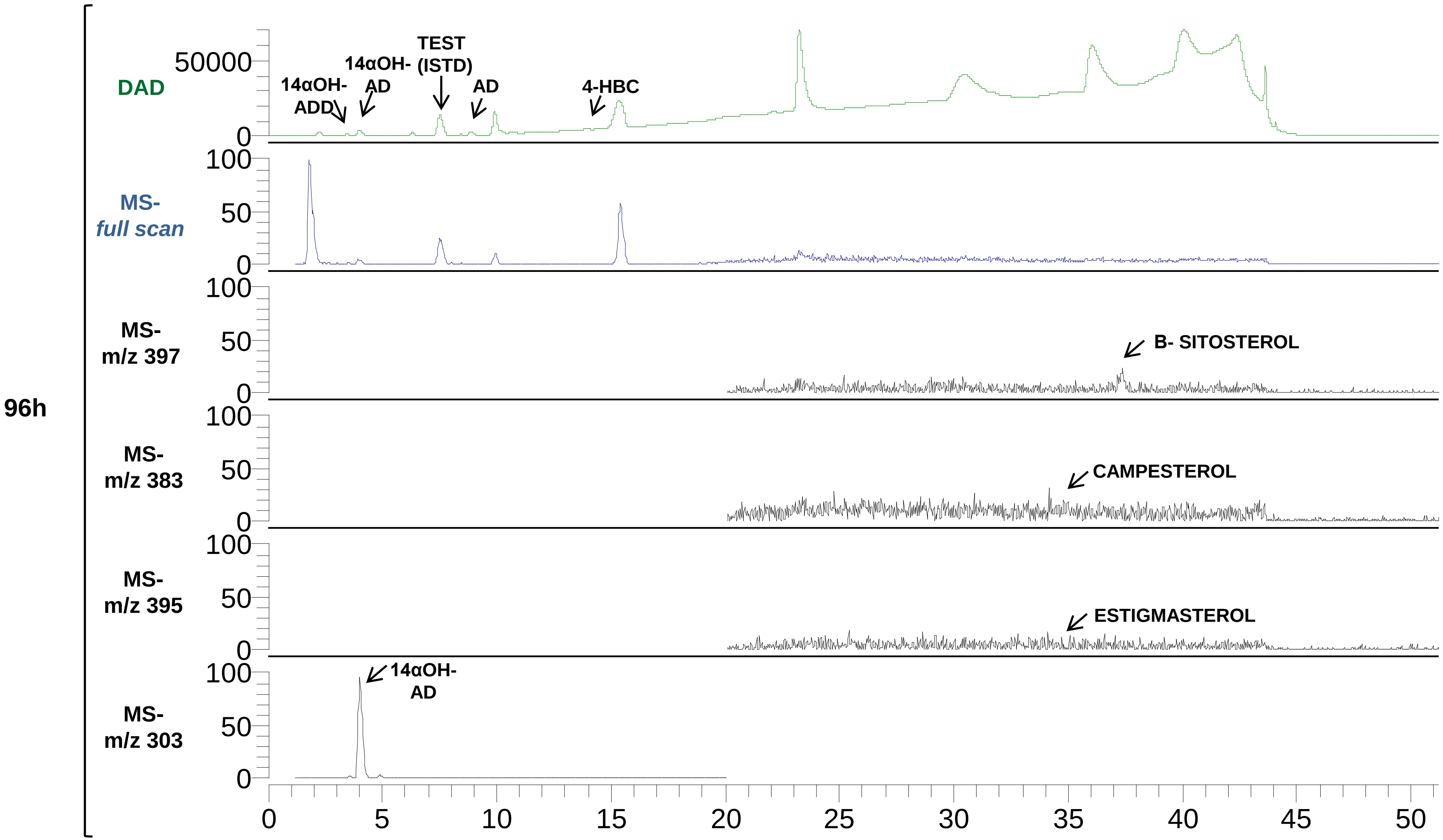
96h
DAD
50000
0
14αOH-
ADD
14αOH-
AD
TEST
(ISTD)
AD
4-HBC
MS-
full scan
100
50
0
MS-
m/z 397
B- SITOSTEROL
MS-
m/z 383
CAMPESTEROL
MS-
m/z 395
ESTIGMASTEROL
MS-
m/z 303
14αOH-
AD
0
5
10
15
20
25
30
35
40
45
50

Figure S4

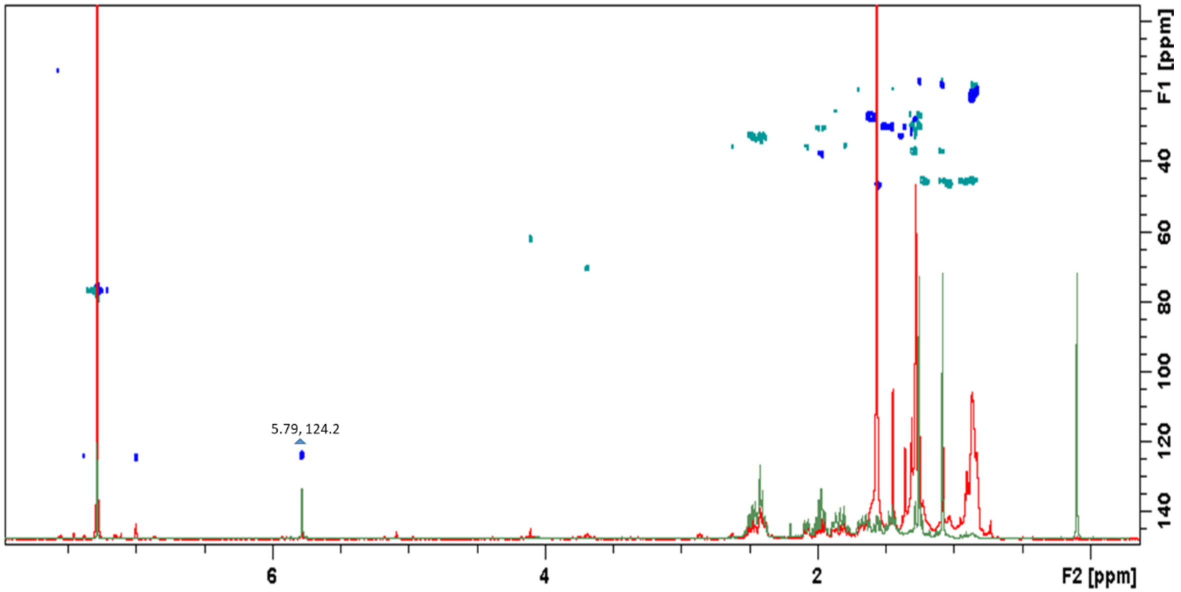

Figure S5

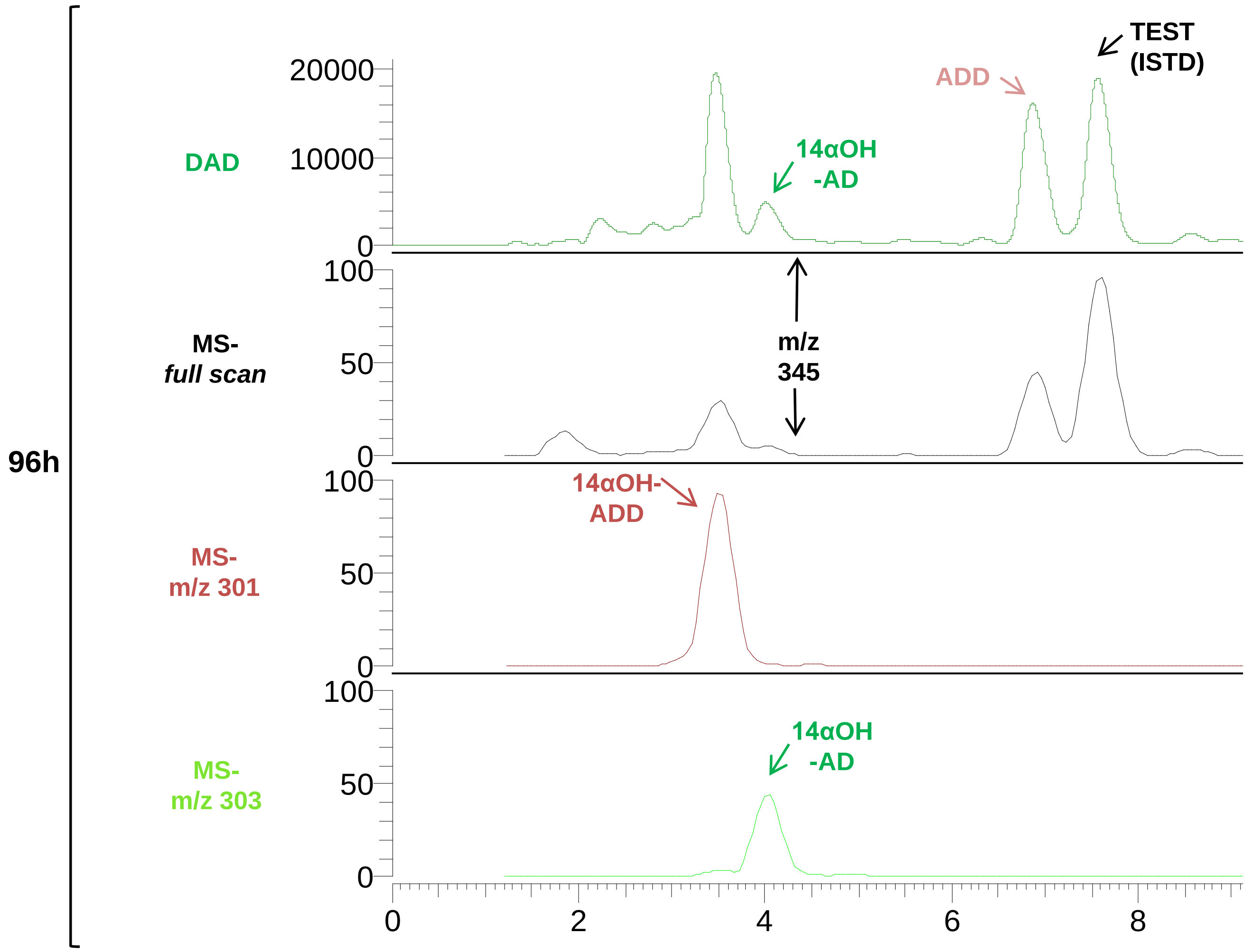

96h
DAD
20000
10000
0
ADD
TEST
(ISTD)
14αOH
-AD
MS-
full scan
100
50
0
m/z
345
MS-
m/z 301
100
50
0
14αOH-
ADD
MS-
m/z 303
100
50
0
14αOH
-AD
0
2
4
6
8

Figure S6

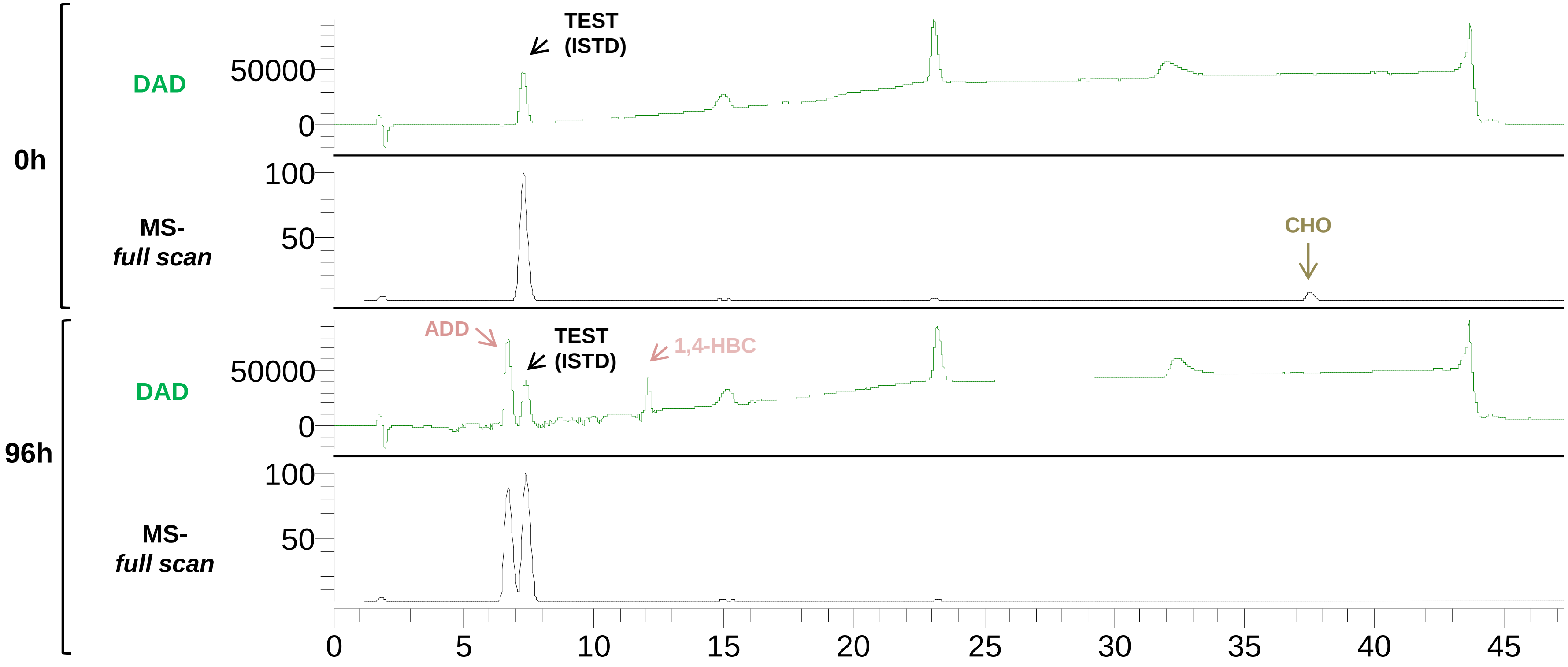

0h
DAD
50000
0
TEST
(ISTD)
MS-
full scan
100
50
CHO
96h
DAD
ADD
TEST
(ISTD)
1,4-HBC
50000
0
MS-
full scan
100
50
0
5
10
15
20
25
30
35
40
45

Figure S7

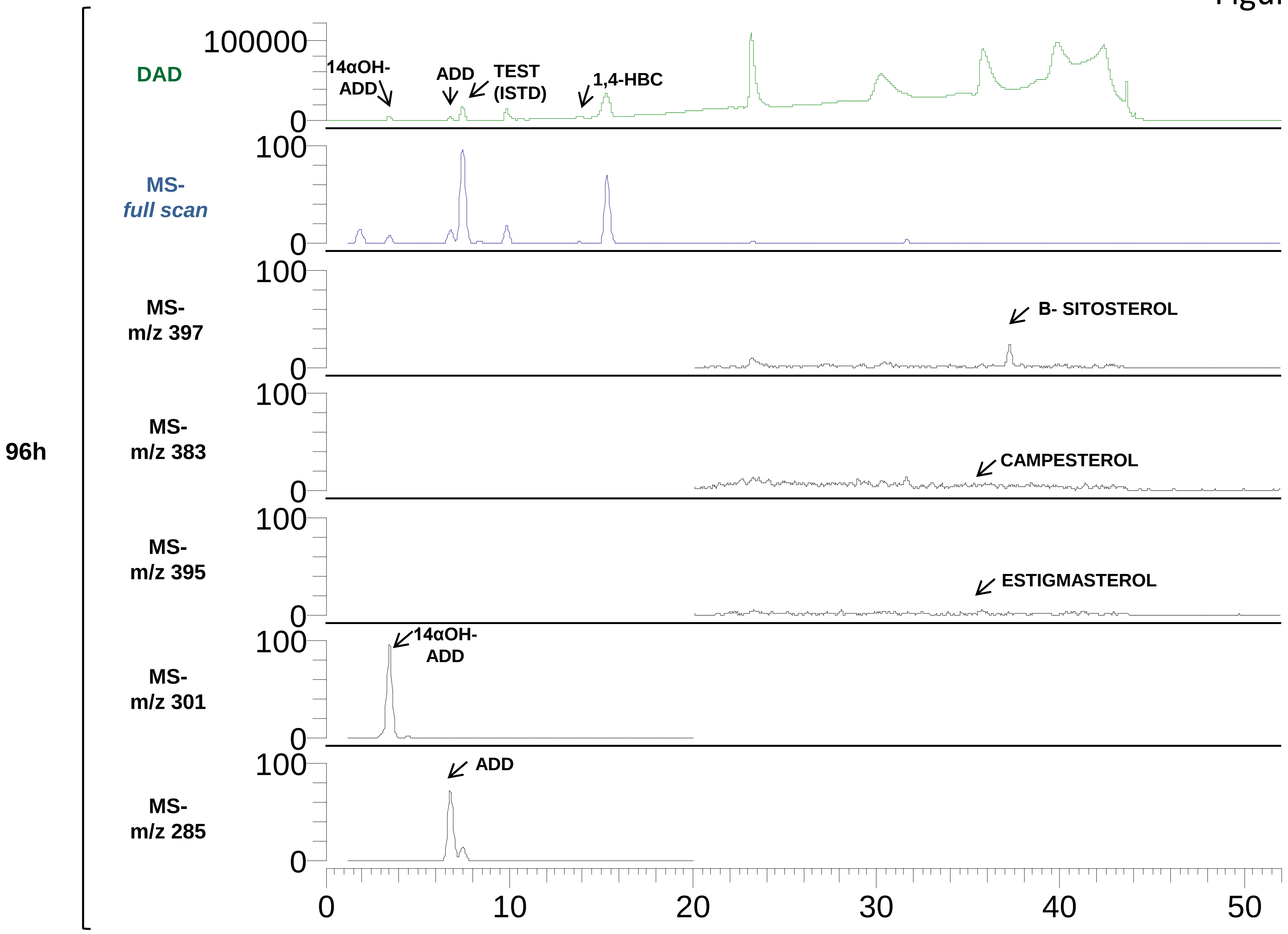
96h
DAD
MS-
full scan
MS-
m/z 397
MS-
m/z 383
MS-
m/z 395
MS-
m/z 301
MS-
m/z 285
100000
0
100
14αOH-
ADD
ADD
TEST
(ISTD)
1,4-HBC
B- SITOSTEROL
CAMPESTEROL
ESTIGMASTEROL
14αOH-
ADD
ADD
0
10
20
30
40
50
